# Supplementary material for: Simulated Microgravity-Induced Changes in SUMOylation and Protein Expression in Saccharomyces cerevisiae
Source: Int J Mol Sci. 2025 Dec 19;27(1):42. doi: 10.3390/ijms27010042 (PMC12786128; doi:10.3390/ijms27010042)
Supplement: Supplementary file 1 [file ijms-27-00042-s001.zip › Figure S1 Cell pellet weights.pdf]

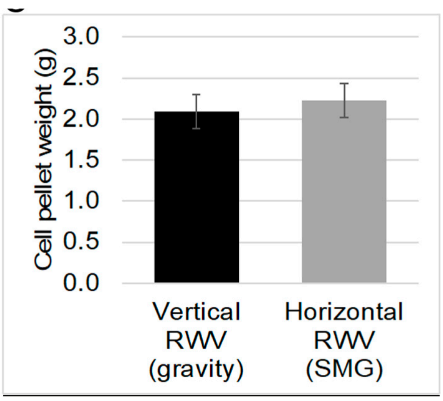

Figure S1. Cell pellet weight averages of *S. cerevisiae* cultured in gravity or simulated microgravity conditions then collected by centrifugation (12 hrs, n = 6). (gravity mean = 2.1±0.21) (SMG mean = 2.2 ± 0.25)
