# Supplementary material for: Simulated Microgravity-Induced Changes in SUMOylation and Protein Expression in Saccharomyces cerevisiae
Source: Int J Mol Sci. 2025 Dec 19;27(1):42. doi: 10.3390/ijms27010042 (PMC12786128; doi:10.3390/ijms27010042)
Supplement: Supplementary file 1 [file ijms-27-00042-s001.zip › Table S2 Differentially expressed proteins in S. cerevisiae proteome with identifiers and qvalues.pdf]

Table S2- Differentially expressed proteins in S. cerevisiae proteome with identifiers and q-values

**UniProt ID**	**Gene name**	**Protein name & description**	**Log_2 fold change (L/H)**	**-Log_{10} T-test p-value**	**T-test q-value**
P32628	RAD23	RAD23_UV excision repair	-2.38	1.65	0.040
P07276	RAD2	RAD2_DNA repair protein	-2.25	2.31	0.056
Q08726	GPN2	GPN2_GPN-loop GTPase2	-2.16	1.41	0.019
P53244	ART5	ART5_Arrestin-related trafficking adaptor 5	-2.03	1.74	0.018
P48237	CCM1	CCM1_Mitochondrial group intron splicing factor	-1.90	1.44	0.024
Q12672	RPL21B	RL21B_60S ribosomal protein L21-B	-1.65	1.35	0.022
P38800	LAM4	LAM4_Membrane-anchored lipid-binding protein	-1.64	1.40	0.044
P36102	PAN3	PAN3_PAB-dependent poly(A)-specific ribonuclease subunit	-1.60	1.80	0.016
P38913	FAD1	FAD1_FAD synthase	-1.38	1.49	0.009
P50111	ZDS1	ZDS1-Regulator of PP2A and Swe1-dependent polarized growth	-1.28	2.09	0.008
P34218	SAS3	SAS3_Histone acetyltransferase	-1.18	2.59	0.019
P38213	DSF2	DSF2_Deletion suppressor of mptFive mutation	-1.11	1.86	0.048
P38850	RTT107	RT107_ Regulator of Ty1 transposition protein 107	-1.09	1.90	0.029
P48360	ARH1	ADRO_Probable NADPH:adrenodoxin oxidoreductase, mitochondrial	-1.04	2.25	0.002
P40026	KRE29	KRE29_DNA repair protein	-1.03	1.40	0.010
Q12321	MED1	MED1_Mediator of RNA polymerase II transcription subunit 1	-0.99	1.77	0.009
Q03233	ADD37	ADD37_A1-proteinase inhibitor-degradation deficient protein 37	-0.97	3.14	0.008

Table S2- Differentially expressed proteins in S. cerevisiae proteome with identifiers and q-values

Q08986	SAM3	SAM3_S-adenylmethionine permease	-0.90	3.28	0.039
P39524	DRS2	ATC3_Probable phospholipid-transporting ATPase	-0.86	1.43	0.021
P33296	UBC9	UBC9_SUMO-conjugating enzyme E2	-0.85	1.46	0.007
Q01649	CIK1	CIK1_Spindle pole body-associated protein	-0.83	2.24	0.023
P42940	CIR1	ETFB_Probable electron transfer flavoprotein subunit beta	-0.79	1.74	0.022
P40970	LCB2	LCB2_Serine palmitoyltransferase 2	-0.75	1.67	0.018
P34909	MOT2	NOT4_General negative regulator of transcription subunit 4	-0.66	1.46	0.024
P53752	YNR066C	YN95_Uncharacterized membrane glycoprotein	-0.64	2.84	0.045
P38198	STU1	STU1_Microtubule associated protein	-0.63	2.05	0.023
P41834	UFE1	UFE1_t-SNARE protein retrograde vesicular traffic	-0.60	1.84	0.023
Q06168	SFH1	SFH1_Chromatin structure-remodeling complex subunit	-0.58	1.97	0.026
P24720	MNE1	MNE1_Mitochondrial mRNA splicing	0.58	2.03	0.018
Q12046	CWC2	CWC2_Pre-mRNA-splicing factor	0.58	1.73	0.025
Q02204	MRPL13	RM1354S_ribosome protein L13, mitochondria	0.64	1.82	0.018
Q08816	YOR352W	TFB6_TFIIH protein complex	0.65	1.41	0.032
P32332	OAC1	OAC1_Mitochondrial oxaloacetate transport protein	0.86	1.66	0.016
